# A Case of Syphilodermata, Accompanied by Peculiar Symptoms

**Published:** 1892-01

**Authors:** J. Henry Dowd

**Affiliations:** 132 Broadway; Buffalo, N. Y.


### A CASE OF SYPHILODERMATA, ACCOMPANIED BY PECULIAR SYMPTOMS.

By J. HENRY DOWD, M. D., Buffalo, N. Y.

In reporting the following case, it is not my object to teach anything either as to the pathology or treatment of this dread disease, but simply to call attention to peculiar symptoms which can arise, also as to how sadly one can be mistaken by simply asking a patient, Did you ever have a hard chancre ? and, receiving a negative reply, try to form a diagnosis, prescribing what you may think appropriate treatment, only to find after a long and persistent course, no benefit has been derived ; where, by a little closer questioning, diagnosis and treatment would have been directed towards the true cause. The symptoms which I wish to call attention to are : (1) The intolerable itching ; (2) the drawing sensation over the affected parts ; and (3) the occurrence of the symptoms mostly at night. In regard to the itching, text-books on syphilis say, almost emphasizing the words, there is an absence of itching, and a practitioner of experience to whom I related this case said : "I thought that an itching skin disease always excluded syphilis." The question may arise, Cannot skin diseases accompanied by itching occur in the subject of syphilis ? Most certainly they can, as I have seen cases of scabies, urticaria, etc., in patients who had and were treated for syphilis. But, on the other hand, could scabies, urticaria, or eczema erythematosium—many of the symptoms of this eruption, both objective and subjective, being present—be cured by the treatment given in this case ?

J. T., single, consulted me for a skin eruption on the scalp, face and body, giving the following history : About the first of last July he noticed spots appearing on his forehead, face, neck and body, these being about the size of a silver five-cent piece, and very itchy. Soon those on the forehead gradually coalesced, until at present there is one patch three inches square, extending into the hair. The most distressing symptoms are the intolerable itching and drawing sensation, which always became aggravated at night, and which he describes "as though the bones were being pulled out." By close questioning I gained a further history : Has had gonorrhea and chancres several times, but always treated himself, and never experiencing any bad after-effects but once, this being about six years ago, when he had four or five sores upon his penis, which he treated in a similar manner as previously, they healing readily ; but in about two weeks afterwards (four weeks after last connection) he discovered a hard lump at the seat of one of the former sores, this being soon followed by swellings in the groin and spots upon the chest, which at first were bright red, but later copper colored (like a copper cent, to use his words). He now consulted a doctor, who treated him, advising him to refrain from drinking, but not saying what was the trouble. As he did not improve in the least, he stopped taking medicine and has remained in the same condition ever since, excepting after an occasional spree, the eruption would break out anew, remaining a week or so and again almost disappearing. In July last, when, after a spree it came out and has staid so, being accompanied by the symptoms above stated.

*Treatment.*—Of course, when the diagnosis of syphilis is sure, the treatment is the same as has been in vogue for years, but it is to the administration of the remedies I wish to call attention. The giving of mercury hypodermically has been practised for years, and of late has been the subject of much discussion in medical literature, the main objection being that abscesses frequently resulted, and which, of course, is a very distressing complication. If the injection has been given according to instructions, it is deep-seated, and often, owing to the patient's condition, is slow of healing. On the other hand, I am convinced that if a clean needle is used, and the solution free from sediment, there is no more danger of an abscess than there would be in giving an injection of morphine, or any substance used hypodermically, and this has been my experience with fifty or sixty cases treated in the U. S. Marine Hospital, also the surgical ward of the Sisters' Hospital, all of which received from eight to twenty injections. I don't wish to infer that all cases should be treated hypodermically, but certain cases demand it and should be so treated, these being chosen according to the symptoms presented, and which I have tried to class under the following heads, which has been my guide in treating syphilis:

I. In all cases where the most rapid effect of the drug is desired.

II. When, by some unexplainable cause, mercury by the mouth disagrees with the patient.

III. When it becomes necessary to discontinue either drug for a time on account of salivation or iodism.

Under the first head can be classed: involvement of eyes, brain, bones, osteocopic or periosteal pains, ulcerations of mucous surfaces, eruptions of the face, dactylitis, etc.

Second. Where mercury, internally, causes diarrhea (which occasionally occurs by the smallest doses), pains in the abdomen, tenderness of the groins without salivation, etc.

Third. Where salivation occurs, and by stopping the mercury the iodide has to be stopped at the same time, and vice-versa. In the case just cited, the treatment was as follows:

℞.—Hydrargr. chlor.corr.................. gr. iii.
Ammon. chlor........................ gr. jss.
Aq. qest........................... ℥ss.
M.—Sig. Fifteen to twenty drops every other day.

℞.—Syr. ferri. iod......................... ℥j

Iod. pot.............................. ʒiii.

Syr. trifol..........................ad. ℥iv.

M.—Sig. One drachm three times a day.

This treatment was continued for sixteen days, the patient being free from itching and the drawing sensation on the fifth day, and at date of writing he was discharged, his eruption having entirely disappeared. Before discharging him I put him on the pill hydrarg. prot. iod., gr. one-sixth, to be continued for four or five months.

132 Broadway.

